# Bladder Cancer Diagnosis and Identification of Clinically Significant Disease by Combined Urinary Detection of Mcm5 and Nuclear Matrix Protein 22

**DOI:** 10.1371/journal.pone.0040305

**Published:** 2012-07-09

**Authors:** John D. Kelly, Tim J. Dudderidge, Alex Wollenschlaeger, Odu Okoturo, Keith Burling, Fiona Tulloch, Ian Halsall, Teresa Prevost, Andrew Toby Prevost, Joana C. Vasconcelos, Wendy Robson, Hing Y. Leung, Nikhil Vasdev, Robert S. Pickard, Gareth H. Williams, Kai Stoeber

**Affiliations:** 1 Department of Pathology and Cancer Institute, University College London, London, United Kingdom; 2 The Royal Marsden National Health Service (NHS) Foundation Trust, London, United Kingdom; 3 Wolfson Institute for Biomedical Research, University College London, London, United Kingdom; 4 Department of Medicine, Imperial College London, London, United Kingdom; 5 Department of Clinical Biochemistry, Addenbrooke’s Hospital, University of Cambridge, Cambridge, United Kingdom; 6 Department of Public Health and Primary Care, Centre for Applied Medical Statistics, University of Cambridge, Institute of Public Health, Cambridge, United Kingdom; 7 Department of Primary Care and Public Health Sciences, King’s College London, London, United Kingdom; 8 Department of Urology, Freeman Hospital, Newcastle upon Tyne, United Kingdom; 9 Beatson Institute for Cancer Research, University of Glasgow, Bearsden, Glasgow, United Kingdom; Johns Hopkins University, United States of America

## Abstract

**Background:**

Urinary biomarkers for bladder cancer detection are constrained by inadequate sensitivity or specificity. Here we evaluate the diagnostic accuracy of Mcm5, a novel cell cycle biomarker of aberrant growth, alone and in combination with NMP22.

**Methods:**

1677 consecutive patients under investigation for urinary tract malignancy were recruited to a prospective blinded observational study. All patients underwent ultrasound, intravenous urography, cystoscopy, urine culture and cytologic analysis. An immunofluorometric assay was used to measure Mcm5 levels in urine cell sediments. NMP22 urinary levels were determined with the FDA-approved NMP22® Test Kit.

**Results:**

Genito-urinary tract cancers were identified in 210/1564 (13%) patients with an Mcm5 result and in 195/1396 (14%) patients with an NMP22 result. At the assay cut-point where sensitivity and specificity were equal, the Mcm5 test detected primary and recurrent bladder cancers with 69% sensitivity (95% confidence interval = 62–75%) and 93% negative predictive value (95% CI = 92–95%). The area under the receiver operating characteristic curve for Mcm5 was 0.75 (95% CI = 0.71–0.79) and 0.72 (95% CI = 0.67–0.77) for NMP22. Importantly, Mcm5 combined with NMP22 identified 95% (79/83; 95% CI = 88–99%) of potentially life threatening diagnoses (i.e. grade 3 or carcinoma in situ or stage ≥pT1) with high specificity (72%, 95% CI = 69–74%).

**Conclusions:**

The Mcm5 immunoassay is a non-invasive test for identifying patients with urothelial cancers with similar accuracy to the FDA-approved NMP22 ELISA Test Kit. The combination of Mcm5 plus NMP22 improves the detection of UCC and identifies 95% of clinically significant disease. Trials of a commercially developed Mcm5 assay suitable for an end-user laboratory alongside NMP22 are required to assess their potential clinical utility in improving diagnostic and surveillance care pathways.

## Introduction

Urothelial cell carcinoma (UCC) of the urinary bladder is the 4th most common cancer in the US, with an estimated 73510 new cases and 14880 deaths from bladder cancer in 2012 [1]. Cystoscopy is the standard method of bladder tumour detection, however it is an invasive, uncomfortable and costly procedure which results in urinary infection in up to 5% of cases [2]. Detection of bladder cancer with a non-invasive tumour marker test could potentially improve the management of the disease by increasing the accuracy and decreasing the morbidity associated with current diagnostic and surveillance pathways. Through reduced frequency of cystoscopies, improvements in patient’s quality of life and cost efficiency could be seen.

Urinary biomarkers for the detection of bladder cancer hold great promise and while numerous markers have regulatory approval none have been accepted as a standard diagnostic procedure [3]. Urinary cytology remains the most widely utilized because of high specificity although poor sensitivity. Novel technologies and biomarkers, however, have the potential to improve diagnostic accuracy, with the most effective diagnostic and surveillance strategies to date utilizing photodynamic cystoscopy and biomarkers [4]. Nuclear matrix protein 22 (NMP22), for example, is a nuclear mitotic apparatus protein that regulates chromatid and daughter cell separation [5], [6] and has emerged as one of the promising urinary biomarkers for UCC [3]. The FDA-approved, laboratory-based quantitative NMP22® Test Kit immunoassay (Matritech, Freiburg, Germany) and a qualitative point-of-care test, NMP22® BladderChek® (Matritech; ® symbol omitted hereafter), are now available for clinical use. However, although urinary NMP22 levels are elevated in bladder cancer, dead and dying urothelial cells in many non-malignant and inflammatory conditions can also release NMP22, thus reducing specificity. Moreover, a wide marked range in test performance has been reported among different studies using NMP22, with sensitivity ranging from 33% to 100% and specificity from 40% to 93% [4].

The constrained accuracy of available biomarkers, along with their expense, has therefore limited introduction of urinary biomarkers into routine clinical practice. Hence there remains an urgent need to identify new biomarkers that might improve diagnostic accuracy, either when used in isolation or in combination with existing biomarker tests [7].

The DNA replication initiation machinery represents a final and critical step in growth control downstream of complex redundant oncogenic signalling pathways and is therefore a potentially attractive diagnostic and therapeutic target [8]. Proteins of the minichromosome maintenance (Mcm) family (Mcm2-7, collectively referred to as MCM), assemble into hexameric complexes that have DNA helicase activity, which is essential for initiation of DNA synthesis [9], [10]. In epithelial-lined organ systems MCM proteins become dysregulated and overexpressed in hyperproliferative dysplastic (preinvasive) and malignant states, [8], [11]–[13]. Indeed the degree of expression of Mcm2 and Mcm5 has been shown to predict recurrence and death in patients with bladder cancer [14]–[16]. Mcm2-7 protein expression in normal epithelium is restricted to the basal stem/transit compartments and is absent from surface layers as cells adopt a fully differentiated phenotype. In premalignant/dysplastic epithelial lesions there is an expansion of the proliferative compartment coupled to arrested differentiation, resulting in the appearance of cycling MCM-positive cells in superficial layers. The detection of exfoliated MCM-positive cells in clinical samples therefore provides a potentially sensitive method for detecting preinvasive and invasive cancers [8], [17], [18]. In a proof-of-principle study we previously showed that elevated Mcm5 levels in cells in urine sediments is predictive of the presence of bladder cancer [19].

The aim of this study was to evaluate Mcm5 as a biomarker for detection of bladder cancer alone, in comparison and in combination with NMP22. The prospective blinded observational trial utilized an immunofluorometric assay to measure Mcm5 and the FDA-approved NMP22 Test Kit.

## Methods

### Study Subjects

Single voided urine specimens were obtained from 1677 patients attending a one stop diagnostic clinic for investigation of haematuria. The diagnosis was established following assessment by cystoscopy, upper urinary tract imaging, urine cytology and culture. Histological confirmation of bladder cancer at subsequent trans-urethral resection was the reference standard and all patients were followed for a period of six months from the time of initial investigations. Patients with a history of recent genito-urinary instrumentation or surgery within the previous two weeks were excluded. Patients with a history of concomitant malignancy or other malignancy within five years prior to study were also excluded. With these exceptions all consecutive patients attending for investigation during the study period were approached for recruitment into the trial.

Urine samples were split equally for: (i) urinalysis and microbiological culture, (ii) cytological analysis, (iii) Mcm5 measurement and (iv) NMP22 measurement. Patients underwent upper urinary tract imaging including ultrasound and intravenous urography. Male patients were examined by digital rectal examination for the presence of clinically detectable prostatic disease. Prostate-specific antigen (PSA) testing was not mandated and PSA levels were checked in a proportion of cases in whom cancer was suspected or who requested the test. If PSA levels were elevated patients were offered trans-rectal ultrasound guided core biopsies of the prostate. Typically all haematuria tests were completed within 24 hours and within two weeks for all patients. Clinical data were entered into a database prospectively prior to Mcm5 and NMP22 analysis. The reference standard for detection of bladder cancer was pathological confirmation following trans-urethral resection.

Urine samples were analyzed in a blinded fashion for Mcm5 detection, NMP22 testing, and cytologic analyses. On completion of the study, we decoded the patient data and compared immunofluorometric Mcm5 signals and NMP22 results with clinical diagnoses based on cystoscopy, biopsy histology, imaging and urine cytology. Staging and grading of malignant tumours was performed by a specialist uro-pathologist using the TNM (tumour-node-metastasis) classification system [20] and the 1973 World Health Organization (WHO) grading system respectively [21].

### Ethics Statement

Ethical approval was obtained from the Joint UCL/UCLH Committees on the Ethics of Human Research (04/Q0502/1), Addenbrooke’s Hospital Ethics Committee (00/236) and the Newcastle and North Tyneside Research Ethics Committee (2002/161). Written informed consent was received from all participants.

### Urine Cytology

Urine samples (50 mL) were centrifuged at 1500 g for 5 min. Cytospin preparations were prepared on poly-L-lysine coated slides using Shandon cytospin tubes and a cytocentrifuge according to the manufacturer’s instructions (Thermo Shandon, Runcorn, UK). Samples were fixed in industrial methylated spirits and stained using the Papanicolaou technique for smears [20]. Specimens were evaluated by a consultant cytologist experienced in uro-pathology. Cytology was scored as positive if atypical or malignant cells were identified.

### NMP22 Assay

NMP22 was measured by enzyme-linked immunosorbent assay (ELISA) using the FDA-approved NMP22 Test Kit produced by Matritech (Freiburg, Germany). The assay run on a Dade Behring BEP 2000 automated ELISA processor (now Siemens Healthcare). All reagents, calibrators and controls were prepared as recommended by the manufacturer. All standards, quality controls and samples were analyzed in duplicate. Results were calculated using the data processing software supplied with the BEP 2000. The lower limit of detection of the assay was found to be 2 U/mL. Samples with concentrations greater than the top standard were repeated after dilution in assay buffer. The between-batch coefficient of variation was 13.3% at a concentration of 11.3 U/mL, 8.8% at 34 U/mL and 9.5% at 65 U/mL. A result for the NMP22 test was available in 1396 patients, including 195 patients (14%) with a urothelial tumour.

### Immunofluorometric Assay to Measure Mcm5 Levels in Urine Sediments

Mcm5 was measured by two-site time-resolved fluorescence immunoassay on the AutoDELFIA analyzer (Perkin Elmer). All standards, quality controls and urine samples were prepared and processed as described [19]. Nunc Maxisorp microtiter plates (Perkin Elmer) were coated with 12A7 mouse anti-human Mcm5 monoclonal antibody [19] at a concentration of 8 mg/L by Dako UK Ltd (Ely, UK). A large batch (approximately 200) of plates were prepared by Dako and used throughout the study. Plates were received pre-blocked and ready for use. A second mouse anti-human Mcm5 monoclonal antibody (4B4) [19] was conjugated with europium by Dako. The europium-labelled antibody was at a concentration of 1.75 mg/mL. HeLa S3 cells were purchased commercially (Health Protection Agency Culture Collections, Porton Down, UK) and the assay was calibrated with processed HeLa cell standards at a concentration of 150000 cells/well. A series of standards spanning the concentration range 150000 to 1500 cells/well were prepared by diluting the stock standard in phosphate buffered saline containing 0.04% SDS and 0.02% sodium azide. Quality control samples containing four different concentrations of HeLa cells were analyzed at the beginning and end of each batch. The protocol for the AutoDELFIA assay was as follows. 50 µL standard, sample or quality control was added (in duplicate) to the antibody-coated microtiter plate along with 100 µL DELFIA multibuffer (Perkin Elmer product code 1380–3614). The plate was incubated for 2.5 h with continuous shaking. The plate was then washed four times with DELFIA wash buffer (Perkin Elmer product code B117-100). Europium-labelled detection antibody 4B4 was diluted 1∶1,800 in DELFIA multibuffer. 100 µL of diluted antibody was added to each well and the plate incubated for a further 4 h with continuous shaking. The plate was then washed six times with DELFIA wash buffer and 200 µL DELFIA enhancement solution (Perkin Elmer product code B118-100) was added to each well. The plate was incubated on a shaker for a further 10 min. The amount of europium in each well was measured on the AutoDELFIA plate reader. Data were automatically transferred to a MultiCalc software package (Perkin Elmer), which was used to generate a calibration curve and calculate the concentration of the unknowns. The lower limit of detection of the assay was found to be 1000 cells/well. Samples with concentrations greater than the top standard were repeated after dilution in the standard dilution buffer. The between-batch coefficient of variation was 11.5% at a concentration of 2648 cells/well and 11.0% at 26382 cells/well. A result for the immunofluorometric Mcm5 test was available in 1564 patients including 210 patients (13%) with a urothelial tumour.

### Statistical Analysis

Sensitivity and specificity characteristics of Mcm5 and NMP22 for the detection of UCC of the bladder are presented as receiver operating characteristic (ROC) curves. The area under the nonparametric ROC curve was used to assess the overall diagnostic performance of each test. Three cut-points were used to demonstrate test performance under different circumstances for Mcm5 as follows: (i) the lower detection limit of the assay where sensitivity of the test was maximal (1000 cells/well) (ii) sensitivity equal to specificity (2150 cells/well) and (iii) 95% specificity (8500 cells/well). Negative predictive value (NPV) and positive predictive value (PPV) were also estimated. An exact 95% confidence interval (CI) for each proportion, including sensitivity, specificity and predictive values for Mcm5 and NMP22, was derived assuming a binomial distribution. The manufacturer’s recommended cut-point for NMP22, 10 U/ml was utilized for all analyses unless otherwise specified.

False positive rates (FPR) for the Mcm5 and NMP22 tests in patients with benign diagnosis were compared with clear normal patients using a Chi-squared test. The Mcm5 and NMP22 values were summarized using medians and interquartile ranges (IQR) and compared with the clear normal patients using the Mann-Whitney U-test. For each biomarker, the ROC analysis was repeated for males and females separately and the areas under the ROC curves were compared using a Chi-squared test with one degree of freedom. ROC analysis was also undertaken to examine the sensitivity of the main results to the exclusion of those with benign disease. The values of the urinary biomarkers for patients with different tumour grades and stages and normal patients were compared using Mann-Whitney U-tests between neighbouring categories, and using the Jonckheere-Terpstra test for trend across grades and stages. The Chi-squared test for linear by linear association was used to assess the evidence for a trend in the false positive rates by increasing tumour grade and stage. The sensitivity determined for urinary cytology was compared with that of the immunofluorometric Mcm5 test using McNemar’s test for paired proportions. The accuracy of a biomarker was defined as the value of sensitivity and specificity where the cut-point provided these to be equal. The accuracy of the two biomarker tests was compared using McNemar’s test. McNemar’s test was also used to compare the sensitivity of cytology with that of each biomarker at cut-points providing the same specificity as observed for cytology. Spearman correlation was used to assess the degree to which the biomarkers were distinctive in UCC case and in normal control groups. All statistical tests were two-tailed, and a 5% level was used to indicate statistical significance.

A multi-ROC analysis [22] was performed to determine the additional performance resulting from using both biomarkers together. In this analysis, NMP22 was kept fixed at the recommended cut-point of 10 U/mL and Mcm5 was included with a varying cut-point. Raised values of either marker could predict positive for UCC. The additional performance of Mcm5 over that obtained from NMP22 (10 U/mL cut-point) was assessed using the nonparametric area under the multi-ROC curve, and assessed for statistical significance using a Chi-squared test with one degree of freedom. In order to demonstrate test performance, Mcm5 was then fixed at the cut-point that provided equal sensitivity and specificity on the multi-ROC curve from using the combined markers. This combination test accuracy was compared with the test accuracy provided by use of NMP22 alone using McNemar’s test.

## Results

### Demographics and Clinical Investigation

The demographic characteristics, mode of presentation, final diagnosis, and tumour grade and stage for the 1677 patients included in this study are summarized in Table 1. The study population was predominantly male (62%) and had a mean age of 60.7 years (standard deviation, 16.3 years). Of those with a recorded presentation, 54% had visible haematuria and 46% had non-visible haematuria. These patients were newly presenting cases, although four patients recruited, later revealed a previous history of UCC. Investigations were omitted in a proportion of cases as follows: cystoscopy was not performed in 20 patients, ultrasound scan in 186 patients and intravenous urography in 223 patients. Urine cytology was unavailable for 109 patients due to insufficient sample collection or, alternatively, because the test was not undertaken. Neither ultrasound scan nor intravenous urography was performed in 77 patients. All patients had a clinical diagnosis attributed to them by their clinician. Data were not formally collected on the adverse effects of standard clinical testing and no adverse effects of urinary testing for Mcm5 or NMP22 were recorded.

**Table 1:** Patient demographics and clinicopathological data.

		n	%	mean	SD	median	IQR
Patients recruited		1677					
Age, years				60.7	16.3	63	49–73
Gender	Male	1040	62				
	Female	637	38				
Bladder/upper tract tumor	Positive	222	13				
	Negative	1455	87				
Grade a	1	26	12				
	2	129	58				
	3 (including CIS)	66	30				
Stage	T0	1455					
	Tx	1					
	Tis	8	4^b^				
	Ta	122	55				
	T1	50	23				
	≥T2	41	18				
Initial referral	Non-visible haematuria	711	46^c^				
	Visible haematuria	851	54				
	Unrecorded	115					

Abbreviations: CIS, carcinoma in situ; IQR, interquartile range; SD, standard deviation.

a n = 221.

b Percentage of patients excluding those with stage T0 and Tx.

c Percentage of recorded cases only.

Following clinical investigation, urinary tract tumours were identified in 222/1677 patients (13%). Nearly all tumours were UCCs, but, investigation also identified one case of adenocarcinoma and two cases of squamous cell carcinoma of the bladder. The UCCs were predominantly bladder tumours, with only seven patients with upper tract tumours. The upper tract UCCs are included alongside the bladder tumours for the analysis reported below. The diagnoses in the remaining patients included other malignancies, benign lesions or cysts of the kidney, benign inflammatory and congenital conditions, urolithiasis, benign prostatic hyperplasia and nephrological diseases. The diagnoses are listed in Table S1. As a component of the diagnostic pathway, urinary cytology had a sensitivity of 9% (95% CI, 5–14%; including atypical cytology as positive), specificity of 88% (95% CI, 86–89%) and PPV of 10% (95% CI, 7–15%).

### Mcm5 and NMP22 Test Performance

**Figure 1:**
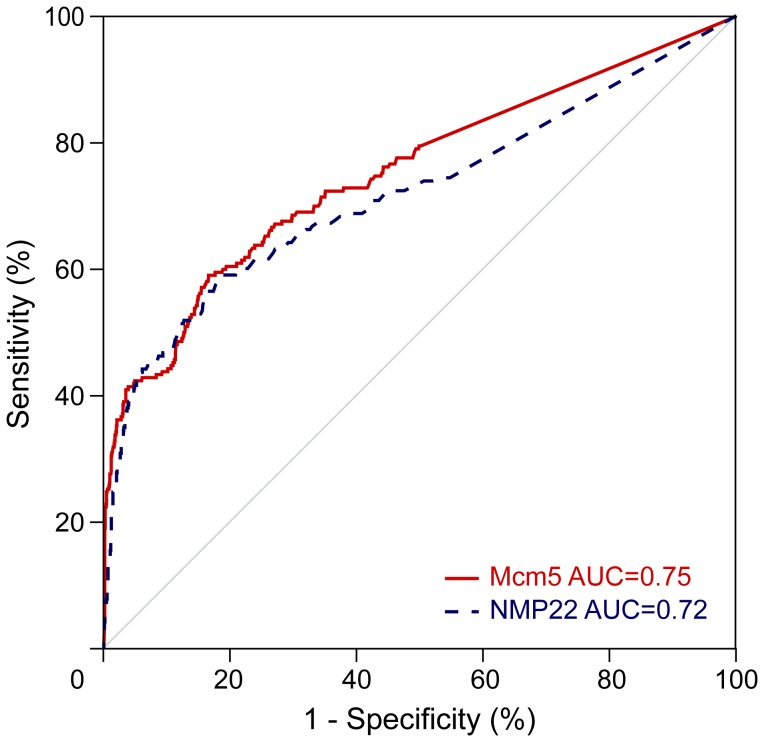
Receiver operating characteristics curves for Mcm5 and NMP22 tests for the detection of bladder cancer in all studied patients with valid test results.

The Mcm5 test discriminated, with high specificity and sensitivity, between patients with and without bladder cancer, as demonstrated by the large area under the ROC curve (AUC) (0.75 [95% CI = 0.71–0.79]) (Figure 1), statistically significantly larger than the area assumed by the null hypothesis (0.5; P<0.001) and based on 210 and 1354 patients respectively with and without UCC.

The sensitivity, specificity, and positive and negative predictive values (PPV and NPV) for Mcm5 are shown in Table 2. The cut-point analysis (cut-points correspond to (i) lower detection limit of the assay; (ii) where sensitivity is equal to specificity, and (iii) specificity of 95% for all patients tested), demonstrated a wide range of test performance levels (Table 2). At the lower detection limit of the assay, the test had 80% (167/210) (95% CI = 73–85%) sensitivity and 20% (167/846) (95% CI = 17–23%) PPV. When sensitivity is equal to specificity, the test had 69% (145/210) (95% CI = 62–75%) sensitivity and 26% (145/565) (95% CI = 22–30%) PPV. At 95% specificity (1286/1354), the test had 42% (89/210) (95% CI = 36–49%) sensitivity and 57% (89/157) (95% CI = 49–65%) PPV.

**Table 2:** Performance of Mcm5 and NMP22 tests for bladder carcinoma detection for all patients with test results available.

Test	Cut-point	n	Sens,% (CI)	Spec,% (CI)	PPV,% (CI)	NPV,% (CI)
Mcm5	1000-cell	1564	80 (73–85)	50 (47–52)	20 (17–23)	94 (92–96)
	2150-cell	1564	69 (62–75)	69 (66–71)	26 (22–30)	93 (92–95)
	8500-cell	1564	42 (36–49)	95 (94–96)	57 (49–65)	91 (90–93)
NMP22	10 U/ml	1396	53 (46–60)	84 (82–86)	36 (30–42)	92 (90–93)
Cytology		1568	9 (5–14)	88 (86–89)	10 (7–15)	87 (85–88)

Abbreviations: CI, 95% confidence interval; NPV, negative predictive value; PPV, positive predictive value; Sens, sensitivity; Spec, specificity.

The NMP22 test discriminated with high specificity and sensitivity as demonstrated by the large AUC (0.72 [95% CI = 0.67–0.77]; null hypothesis [0.5; P<0.001] (Figure 1)) and based on 195 and 1201 patients respectively with and without UCC. The sensitivity, specificity, and positive and negative predictive values for NMP22 at the recommended 10 U/ml cut-point are shown in Table 2. Sensitivity was 53% (104/195) (95% CI = 46–60%) and PPV 36% (104/291) (95% CI = 30–42%).

In order to assess the performance of the test in patients with different stages and grades of disease the True Positive Rate (TPR) was calculated for Mcm5 (at the different cut-points), NMP22 and cytology for muscle invasive vs non-muscle invasive (Table S2) and across grades (Table S3). Test performance improved for all tests in higher stage and grade categories.

Where the specificity of NMP22 (cut-point 12.1) was the same as that of positive cytology (88%; 989/1128), the sensitivity of NMP22 was significantly higher (P<0.001) (51%; 91/177 versus 8%; 14/177). Where the specificity of Mcm5 (5150-cell cut-point) was the same as that of cytology (87%; 1109/1271) the sensitivity of Mcm5 was significantly higher (P<0.001) (52%; 100/193 versus 9%; 17/193).

### Biomarker False Positive Analysis

False positives were found in 400/1301 (31%) of clear normal and benign diagnosis patients with the Mcm5 test at the 2150-cell cut-point. There was a significantly higher rate of false positive results in female patients, 38% (200/520) compared to males 26% (200/781) (P<0.001). Urinary Mcm5 levels were also significantly higher in normal/benign females compared to males (median 1560 cells/well [IQR = <1000–3675 cells/well] vs median <1000 cells/well [IQR = <1000–2180 cells/well], P<0.001). Furthermore, compared to normal patients, those with, urinary calculi had a significantly higher false positive rate (44% [47/106] vs 30% [201/661], P = 0.004) and higher urinary levels of Mcm5 protein (median 1840 cells/well [<1000–3963 cells/well] vs 1040 cells/well [<1000–2645 cells/well], P<0.001; Table 3). There was no evidence of an association between the false positive rate and any of the other benign groups including inflammatory conditions and benign prostatic hyperplasia. In the clear normal and benign patient groups there were no significant differences (P = 0.99) in NMP22 levels between males and females. A raised NMP22 signal and increased false positive rate was observed for those patients with urinary tract infections (FPR: 22% vs 11%, P = 0.001; median NMP22 result: 3.35 U/mL vs 2.2 U/mL, P<0.001) and urinary calculi (FPR: 23% vs 11%, P = 0.001; NMP22∶2.55 U/mL vs 2.2 U/mL, P = 0.047) (Table 3).

**Table 3:** False positive rates for the Mcm5 and NMP22 tests across benign conditions.

Test	Benign condition	n	Test value, med (IQR)	P^a^	FPR, %^b^	P^c^
Mcm5^d^	Clear normal	661	1040 (<1000–2645)		30	
	BPH	132	<1000 (<1000–2438)	0.056	27	0.37
	Calculi	106	1840 (<1000–3963)	<0.001	44	0.004
	Nephrological	40	<1000 (<1000–2448)	0.25	25	0.47
	Prostatitis	14	<1000 (<1000–3433)	0.37	29	0.88
	Urethral stricture	21	1100 (<1000–2620)	0.94	29	0.86
	UTI	246	<1000 (<1000–2823)	0.84	30	0.92
	Other	81	1110 (<1000–2855)	0.68	28	0.71
NMP22^e^	Clear normal	589	2.20 (<2.00–5.30)		11	
	BPH	110	<2.00 (<2.00–5.60)	0.96	13	0.65
	Calculi	96	2.55 (<2.00–9.48)	0.047	23	0.001
	Nephrological	38	2.05 (<2.00–6.43)	0.93	11	0.9
	Prostatitis	13	<2.00 (<2.00–3.50)	0.14	0	0.2
	Urethral stricture	18	2.85 (<2.00–6.83)	0.36	17	0.47
	UTI	222	3.35 (<2.00–8.63)	<0.001	22	<0.001
	Other	68	2.80 (<2.00–8.03)	0.018	24	0.004

For each subgroup, only those patients with a test results were considered.

Abbreviations: BPH, benign prostatic hyperplasia; FPR, false positive rate; IQR, interquartile range; med, median; UTI, urinary tract infection.

a Mann-Whitney test, comparison of test value with normal.

b False positive rate determined using 2150-cell cut-point for Mcm5 test and 10 U/mL cut-point for NMP22 test.

c Chi-squared test, comparison of false positive rate with Normal group.

d Excludes 53 “other cancers” of the 1354 patients without UCC having an Mcm5 test value.

e Excludes 47 “other cancers” of the 1201 patients without UCC having an NMP22 test value.

The ROC analysis for Mcm5 and NMP22 was repeated observing the results in all males and females (Table S4 and Figure S1). There were no significant differences in AUC values for Mcm5 between males and females (P = 0.76), but there was a significant difference in the NMP22 AUC value between males and females (AUC 0.69 for males vs 0.80 for females, P = 0.025), apparently related to the greater NMP22 sensitivity in females.

### Biomarker False Negative Analysis

Table 4 and Table S5 show the false negative rates of urinary Mcm5 and NMP22 grouped by tumour grade and stage. There was evidence of a decreasing trend in the false-negative rate with increasing tumour grade and stage for both urinary biomarkers. For grades 1, 2 and 3 respectively, the false negative rates for urinary Mcm5 at the 2150-cell cut-point were 52% (95% CI = 31–73%), 37% (95% CI = 28–46%) and 11% (95% CI = 4–22%; trend P<0.001). For NMP22 at the 10 U/mL cut-point, the corresponding false negative rates were 80% (95% CI = 59–93%), 49% (95% CI = 40–59%) and 25% (95% CI = 14–40%; trend P<0.001). Similar trends were observed for tumour stage. A significant decrease in the amplitude of the Mcm5 and NMP22 signal with lower tumour grade and stage was observed, in keeping with the increasing false negative rates observed for these groups (Table 4 and Table S5).

### Combined Biomarker Multi-ROC Analysis

There were 183 bladder UCCs and 1100 normal patients with assay data available for both urinary markers. For these patients, an Mcm5 cut-point of 2180 cells/well provided equal sensitivity and specificity of 71% (130/183 and 777/1100), and for NMP22 a cut-point of 4.6 U/mL provided equal sensitivity and specificity of 67% (123/183 and 742/1100). Although there was modestly greater performance of Mcm5 compared with NMP22 in terms of accuracy (71% versus 67%, difference of 3.3%, 95% CI = −0.2–6.7%), this difference was not statistically significant (McNemar’s test: P = 0.067).

The Spearman correlation coefficients between Mcm5 and NMP22 were moderately high (rho = 0.54) for UCC cases and negligible (rho = 0.08) for the normal group, indicating potential for the biomarkers to provide distinct roles within a combination. On the basis of multi-ROC analysis, the immunofluorometric Mcm5 test, in combination with NMP22 at the recommended 10 U/mL cut-point, offers a statistically significant increase in performance (P<0.001) compared with NMP22 alone at the recommended cut-point (area under multi-ROC curve = 0.65, 95% CI = 0.58–0.71). As a demonstration, if either NMP22 exceeds 10 U/mL or Mcm5 exceeds the 4200-cell cut-point, this combination provides sensitivity (131/183) and specificity (789/1100) both equal to 72%, which indicates the improvement over use of NMP22 alone where sensitivity (123/183) and specificity (742/1100) both equal 67% (72% versus 67%, difference = 4.3% [95% CI = 1.5–7.0%], McNemar’s test P = 0.002). In combination with NMP22 at 10 U/mL the MCM5 test removes false negatives from the NMP22 test, offering an improvement from the 54% sensitivity of NMP22 alone to 75% sensitivity with 65% specificity (2800-cell cut-point), or to 80% sensitivity with 58% specificity (1900-cell cut-point), or to maximal sensitivity of 85% with 45% specificity (1000-cell cut-point).

In the combination analysis, with NMP22 (10 U/mL cut-point) and Mcm5 (4200-cell cut-point, where sensitivity and specificity are equal), 100% (31/31) of muscle invasive cancers (i.e. stage ≥ T2), 93% (40/43) of pT1 tumours and 53% (54/102) of pTa tumours were detected. The total number of patients with carcinoma in situ was low and 86% (6/7) were detected. Grade 1 disease was identified in 46% (10/22), grade 2 disease in 64% (68/106) and grade 3 disease in 96% (53/55) of cases (including 6/7 cases of carcinoma in situ). Importantly, Mcm5 combined with NMP22 identified 95% (79/83, 95% CI = 88–99%) of potentially life threatening diagnoses (i.e. grade 3 or CIS or stage ≥pT1) with high specificity (72%, 95% CI = 69–74%).

## Discussion

In an earlier proof-of-concept study we showed that elevated Mcm5 levels in urine cell sediments are highly predictive of bladder cancer [19]. The prospective blinded observational trial reported here, involving a large patient cohort, confirms our initial observations that Mcm5 is a sensitive and specific biomarker for detection of UCC. Importantly, through multi-ROC analysis, we show here that the Mcm5 test, in combination with NMP22 at the established cut-point 10 U/mL, enhances diagnostic accuracy over NMP22 in isolation and identifies nearly all potentially life threatening disease.

**Table 4:** Comparison of Mcm5 and NMP22 test performance across grade and stage.

Test			n	Test value, med (IQR)	P^a^	P^b^	P^c^	FNR, % (CI)	P^a^	P^b^	P^c^
Mcm5^d^	Normal		1354	1015 (<1000–2790)				69 (66–71)			
	Grade^e^	1	23	1300 (<1000–5310)	0.14			52 (31–73)	0.085		
		2	123	4070 (1170–12900)	<0.001	0.041		37 (28–46)	<0.001	0.16	
		3	55	40900 (5800–122000)	<0.001	<0.001	<0.001	11 (4–22)	<0.001	<0.001	<0.001
	Stage^e^	pTa	115	2590 (<1000–5710)	<0.001			46 (37–56)	<0.001		
		pT1	48	39000 (5818–136250)	<0.001	<0.001		10 (3–23)	<0.001	<0.001	
		≥pT2	38	19850 (7150–65800)	<0.001	0.28	<0.001	13 (4–28)	<0.001	0.69	<0.001
NMP22^f^	Normal		1201	2.40 (<2.00–6.30)				84 (82–86)			
	Grade^g^	1	25	<2.00 (<2.00–8.45)	0.31			80 (59–93)	0.55		
		2	112	10.20 (2.68–39.83)	<0.001	<0.001		49 (40–59)	<0.001	0.005	
		3	51	62.50 (9.90–145.50)	<0.001	<0.001	<0.001	25 (14–40)	<0.001	0.005	<0.001
	Stage^g^	pTa	109	6.00 (<2.00–24.50)	<0.001			62 (53–71)	<0.001		
		pT1	45	31.30 (5.70–125.90)	<0.001	<0.001		29 (16–44)	<0.001	<0.001	
		≥pT2	34	70.65 (22.68–258.50)	<0.001	0.099	<0.001	21 (9–38)	<0.001	0.40	<0.001

Abbreviations: CI, 95% confidence interval; IQR, interquartile range; med, median; FNR, false negative rate.

a Mann-Whitney test (for Test value) or Chi-squared test (for FNR), comparison with Normal group.

b Mann-Whitney test (for Test value) or Chi-squared test (for FNR), comparison with previous, i.e. Grade 2 vs Grade 1, Grade 3 vs Grade 2.

c Jonckheere-Terpstra test for trend (for Test value) or Chi-squared test for linear by linear association, across Grade or Stage, excluding Normal group.

d Data analysis using 2150-cell cut-point for Mcm5 test.

e Excludes 8 CIS and 1 adenocarcinoma from 210 UCC cases having an MCM5 test value.

f Data analysis using 10 U/mL cut-point for NMP22 test.

g Excludes 7 CIS from the 195 UCC cases having an NMP22 test value.

Despite numerous studies over the last decade, the reported accuracy of the NMP22 test is highly variable. Many of the earlier studies recruited small to moderate numbers of subjects and reported high sensitivities and specificities, above 80% [23]–[26]. However, a wide range in test performance has been observed in more recent studies with sensitivity ranging from 33% to 100% and specificity from 40% to 93% [4]. A pooled analysis including more recent trials suggests a sensitivity of around 68% and a specificity of 79% [4]. A recent large multi-institutional international trial revealed a marked variability in the performance of the NMP22 test across participating institutions with sensitivity and specificity ranging from 36% to 86% and 50% to 94% respectively [27]. Variability has been attributed to many confounding factors including biological, analytical and epidemiological variables and methodological bias.

Our study represents the largest prospective observational trial ever undertaken using the NMP22 urinary biomarker. Notably, the performance at the 10 U/mL cut-point, with a sensitivity of 53% and specificity of 84%, is somewhat below that reported in the pooled analysis, but almost identical to the diagnostic performance reported in the Matritech supported large patient cohort trials using the NMP22 point-of-care proteomic assay [4], [28]. Interestingly we observed significantly greater diagnostic accuracy of NMP22 in females compared to males. Gender differences in NMP22 test performance have been previously noted [29], [30] however our data represent the largest study of this question and clearly establishes a clinically meaningful difference in test performance.

The analysis of false positive Mcm5 results in this study also revealed an unexpected difference between the male and female groups. The overall false positive rate in females was 38% compared to 26% in males. Rather than being related to benign pathology, the difference was most marked in the clear normal group. These findings require further investigation. Possible causes could be fungal contamination by vaginal flora (e.g. *Candida* species) or mixing of menstrual endometrial contaminants in samples, both sources of extraneous MCM expressing cells. Patients with urinary calculi had the highest incidence of false positive Mcm5 results (44%). As previously reported, higher false positive rates are expected in patients with urinary calculi due to the associated mucosal injury, which exposes the underlying MCM expressing transit amplifying compartment of the transitional epithelium to the urinary tract [8], [19], [31]. However, exclusion of patients with calculi from the ROC analysis did not make a significant improvement to the overall performance, presumably because they were a relatively small group (data not shown). Notably, other benign conditions such as urinary tract infection or benign prostatic hyperplasia were not associated with false positive Mcm5 results, in keeping with our proof-of-concept study [19]. In contrast to the Mcm5 test, false positive NMP22 results were linked to urinary tract infection. The different aetiologies for false positives with Mcm5 and NMP22 may account for the improved performance observed when combining the two urinary biomarkers.

Decreasing urinary Mcm5 and NMP22 signals were observed with lower stage and grade of UCC, and this was associated with an increasing false negative rate for both tests. Expression of MCM proteins in bladder cancer is closely linked to grade [15], [16] and thus this trend is expected. The trend is also explained by the less spontaneous shedding of tumour cells seen in lower grade lesions due to stronger cell-cell and cell-matrix attachments. Commercial development of the Mcm5 test is currently underway and improvements in the assay design to enhance sensitivity are planned and thus reduced false negative rates in early stage, well-differentiated tumours are anticipated. The trend for higher grade and stage tumours to exhibit higher Mcm5 levels could provide a useful predictive clinical role e.g. to target imaging and rigid cystoscopic diagnostic procedures for high risk patients identified by urinary Mcm5. This potential role requires further study.

Current routine initial investigations for haematuria or other symptoms suggestive of bladder cancer include flexible cystoscopy and rigid white light cystoscopy. However an estimated 10–40% of tumours can be missed due to poor visualization as a result of inflammatory conditions or bleeding and flat urothelial lesions such as severe dysplasia and carcinoma in situ [32]–[34]. Photodynamic diagnosis is a technique that can enhance tumour detection but its increased sensitivity is associated with higher false positive rates leading to additional unnecessary investigations, biopsies and thus increased cost [35]. Urinary biomarkers also have potential to enhance tumour detection and identify tumours not visualized during initial endoscopy. A systematic review of the clinical effectiveness and cost-effectiveness of photodynamic diagnosis, cytology and urine biomarkers, including FISH, ImmunoCyt and NMP22, for detection and surveillance of bladder cancer has recently been undertaken [4]. Urinary cytology had the lowest pooled sensitivity of the markers studied at 44% although specificity was highest at 96%. The range of reported sensitivity for cytology was 7–100%. Thus while our study reports low sensitivity for cytology this is not a unique finding. Pooled analyses performed by Mowatt et al showed similar diagnostic performance with NMP22 (sensitivity 84%, specificity 75%) and FISH (sensitivity 76%, specificity 85%) with ImmunoCyt slightly outperforming them (sensitivity 84% specificity 75%) [4]. Notably, of eight diagnosis and follow-up strategies included in a probabilistic sensitivity analysis using combinations of photodynamic diagnosis, flexible cystoscopy, white light cystoscopy, cytology and urinary biomarkers, four were associated with around a 20% chance of being considered cost-effective. Three of these four strategies involved the use of either a biomarker or photodynamic diagnosis. Other urinary markers of bladder cancer such as Survivin [36], various urinary micro RNAs [37], [38] and epigenetic markers [38] have also been shown to have great potential as diagnostic markers with sensitivity/specificity reported >90%. As yet these markers have not been evaluated in large-scale blinded observational studies thus the initial findings from these carefully controlled trials should be interpreted with caution.

In this study, the performance of Mcm5 is similar to that of NMP22 and both markers are significantly more accurate than urinary cytology. It also outperforms the performance of cytology from studies combined in a recent systematic review [4]. The performance of Mcm5 falls below the reported accuracy of ImmunoCyt and some other novel approaches detailed above. It is worth noting that Mcm5 initially demonstrated an AUC of 0.93 in our earlier smaller study and it remains to be seen if the performance of Survivin and other novel markers is reproducible in large studies. Our current data suggest that an Mcm5 assay commercially developed for an end-user laboratory in combination with NMP22 could be used to modify diagnostic and surveillance care pathways to enhance the diagnostic accuracy in those at high risk (e.g. newly presenting visible haematuria patient) and reduce morbidity and cost of testing in low risk patients (e.g. newly presenting non-visible haematuria patient without a known risk factor or a patient with prior low grade non-muscle invasive tumour). Trials to evaluate modified against standard diagnostic pathways using a commercialised assay are currently in preparation.

In conclusion, we have demonstrated that immunofluorometric detection of Mcm5 in urine sediments is a sensitive and specific diagnostic test for bladder cancer. The test detects bladder cancers of all stages and grades. Through evaluation of different assay cut-points there could be a role for predicting high grade and stage disease. Importantly, urinary Mcm5 in combination with the urinary NMP22 measured with the FDA-approved Matritech NMP22® Test Kit, identifies nearly all life threatening disease.

## Supporting Information

Figure S1 **Receiver operating characteristics curves for the (A) Mcm5 and (B) NMP22 tests for detection of bladder cancer in male and female patients.**
(TIF)

Table S1 **Patient diagnoses.**
(PDF)

Table S2 **True positive rate of Mcm5 and NMP22 tests and cytology, across stage, for bladder carcinoma detection.**
(PDF)

Table S3 **True positive rate of Mcm5 and NMP22 tests and cytology, across grade, for bladder carcinoma detection.**
(PDF)

Table S4 **Comparison of Mcm5 and NMP22 test performance in male and female patients.**
(PDF)

Table S5 **True and false negative rates of the Mcm5 and NMP22 tests, by tumour grade and stage.**
(PDF)

## References

[1] American Cancer Society (2012). Cancer Facts & Figures 2012.. http://www.cancer.org/acs/groups/content/epidemiologysurveilance/documents/document/acspc-031941.pdf.

[2] Almallah YZ, Rennie CD, Stone J, Lancashire MJ (2000). Urinary tract infection and patient satisfaction after flexible cystoscopy and urodynamic evaluation.. Urology.

[3] Babjuk M, Oosterlinck W, Sylvester R, Kaasinen E, Bohle A (2012). Guidelines on Non-muscle-invasive Bladder Cancer (TaT1 and CIS). Uroweb 2012.. http://www.uroweb.org/gls/pdf/05_TaT1_Bladder_Cancer_LR%20March%2013th%202012.pdf.

[4] Mowatt G, Zhu S, Kilonzo M, Boachie C, Fraser C (2010). Systematic review of the clinical effectiveness and cost-effectiveness of photodynamic diagnosis and urine biomarkers (FISH, ImmunoCyt, NMP22) and cytology for the detection and follow-up of bladder cancer.. Health Technol Assess.

[5] Compton DA, Cleveland DW (1993). NuMA is required for the proper completion of mitosis.. J Cell Biol.

[6] Shelfo SW, Soloway MS (1997). The role of nuclear matrix protein 22 in the detection of persistent or recurrent transitional-cell cancer of the bladder.. World J Urol.

[7] Gaston KE, Grossman HB (2010). Proteomic assays for the detection of urothelial cancer.. Methods Mol Biol.

[8] Williams GH, Stoeber K (2007). Cell cycle markers in clinical oncology.. Curr Opin Cell Biol.

[9] Machida YJ, Hamlin JL, Dutta A (2005). Right place, right time, and only once: replication initiation in metazoans.. Cell.

[10] Remus D, Diffley JF (2009). Eukaryotic DNA replication control: lock and load, then fire.. Curr Opin Cell Biol.

[11] Williams GH, Romanowski P, Morris L, Madine M, Mills AD (1998). Improved cervical smear assessment using antibodies against proteins that regulate DNA replication.. Proc Natl Acad Sci U S A.

[12] Going JJ, Keith WN, Neilson L, Stoeber K, Stuart RC (2002). Aberrant expression of minichromosome maintenance proteins 2 and 5, and Ki-67 in dysplastic squamous oesophageal epithelium and Barrett’s mucosa.. Gut.

[13] Blow JJ, Gillespie PJ (2008). Replication licensing and cancer–a fatal entanglement?. Nat Rev Cancer.

[14] Burger M, Denzinger S, Hartmann A, Wieland WF, Stoehr R (2007). Mcm2 predicts recurrence hazard in stage Ta/T1 bladder cancer more accurately than CK20, Ki67 and histological grade.. Br J Cancer.

[15] Korkolopoulou P, Givalos N, Saetta A, Goudopoulou A, Gakiopoulou H (2005). Minichromosome maintenance proteins 2 and 5 expression in muscle-invasive urothelial cancer: a multivariate survival study including proliferation markers and cell cycle regulators.. Hum Pathol.

[16] Kruger S, Thorns C, Stocker W, Muller-Kunert E, Bohle A (2003). Prognostic value of MCM2 immunoreactivity in stage T1 transitional cell carcinoma of the bladder.. Eur Urol.

[17] Eward KL, Obermann EC, Shreeram S, Loddo M, Fanshawe T (2004). DNA replication licensing in somatic and germ cells.. J Cell Sci.

[18] Barkley LR, Hong HK, Kingsbury SR, James M, Stoeber K (2007). Cdc6 is a rate-limiting factor for proliferative capacity during HL60 cell differentiation.. Exp Cell Res.

[19] Stoeber K, Swinn R, Prevost AT, De Clive-Lowe P, Halsall I (2002). Diagnosis of genito-urinary tract cancer by detection of minichromosome maintenance 5 protein in urine sediments.. J Natl Cancer Inst.

[20] UICC International Union Against Cancer (2009). TNM Classification of Malignant Tumours Seventh Edition.. Indianapolis: Wiley-Blackwell.

[21] Mostofi FK, Sobin LH, Torloni H (1973). Histological typing of urinary bladder tumours.. Geneva: World Health Organization.

[22] Shultz EK (1995). Multivariate receiver-operating characteristic curve analysis: prostate cancer screening as an example.. Clin Chem.

[23] Saad A, Hanbury DC, McNicholas TA, Boustead GB, Morgan S (2002). A study comparing various noninvasive methods of detecting bladder cancer in urine.. BJU Int.

[24] Sanchez-Carbayo M, Herrero E, Megias J, Mira A, Soria F (1999). Evaluation of nuclear matrix protein 22 as a tumour marker in the detection of transitional cell carcinoma of the bladder.. BJU Int.

[25] Zippe C, Pandrangi L, Agarwal A (1999). NMP22 is a sensitive, cost-effective test in patients at risk for bladder cancer.. J Urol.

[26] Ponsky LE, Sharma S, Pandrangi L, Kedia S, Nelson D (2001). Screening and monitoring for bladder cancer: refining the use of NMP22.. J Urol.

[27] Shariat SF, Marberger MJ, Lotan Y, Sanchez-Carbayo M, Zippe C (2006). Variability in the performance of nuclear matrix protein 22 for the detection of bladder cancer.. J Urol.

[28] Grossman HB, Soloway M, Messing E, Katz G, Stein B (2006). Surveillance for recurrent bladder cancer using a point-of-care proteomic assay.. JAMA.

[29] Redorta JP, Pascual M, Cosentino M, Caicedo JI, Rodriguez O (2011). Effect of retention time on NMP22 bladder check assay results in voided urine.. Nephro-Urol Mon.

[30] Lotan Y, Shariat SF (2008). Impact of risk factors on the performance of the nuclear matrix protein 22 point-of-care test for bladder cancer detection.. BJU Int.

[31] Ayaru L, Stoeber K, Webster GJ, Hatfield AR, Wollenschlaeger A (2008). Diagnosis of pancreaticobiliary malignancy by detection of minichromosome maintenance protein 5 in bile aspirates.. Br J Cancer.

[32] Zaak D, Kriegmair M, Stepp H, Stepp H, Baumgartner R (2001). Endoscopic detection of transitional cell carcinoma with 5-aminolevulinic acid: results of 1012 fluorescence endoscopies.. Urology.

[33] Schneeweiss S, Kriegmair M, Stepp H (1999). Is everything all right if nothing seems wrong? A simple method of assessing the diagnostic value of endoscopic procedures when a gold standard is absent.. J Urol.

[34] Kriegmair M, Baumgartner R, Knuchel R, Stepp H, Hofstadter F (1996). Detection of early bladder cancer by 5-aminolevulinic acid induced porphyrin fluorescence.. J Urol.

[35] Gakis G, Kruck S, Stenzl A (2010). Can the burden of follow-up in low-grade noninvasive bladder cancer be reduced by photodynamic diagnosis, perioperative instillations, imaging, and urine markers?. Curr Opin Urol.

[36] Ku JH, Godoy G, Amiel GE, Lerner SP (2012). Urine survivin as a diagnostic biomarker for bladder cancer: a systematic review.. BJU Int.

[37] Hanke M, Hoefig K, Merz H, Feller AC, Kausch I (2010). A robust methodology to study urine microRNA as tumor marker: microRNA-126 and microRNA-182 are related to urinary bladder cancer.. Urol Oncol.

[38] Wang G, Chan ES, Kwan BC, Li PK, Yip SK (2012). Expression of microRNAs in the Urine of Patients With Bladder Cancer.. Clin Genitourin Cancer.

