# Supplementary figures and images for: Bladder Cancer Diagnosis and Identification of Clinically Significant Disease by Combined Urinary Detection of Mcm5 and Nuclear Matrix Protein 22
Source: PLoS One. 2012 Jul 9;7(7):e40305. doi: 10.1371/journal.pone.0040305 (PMC3392249; doi:10.1371/journal.pone.0040305)

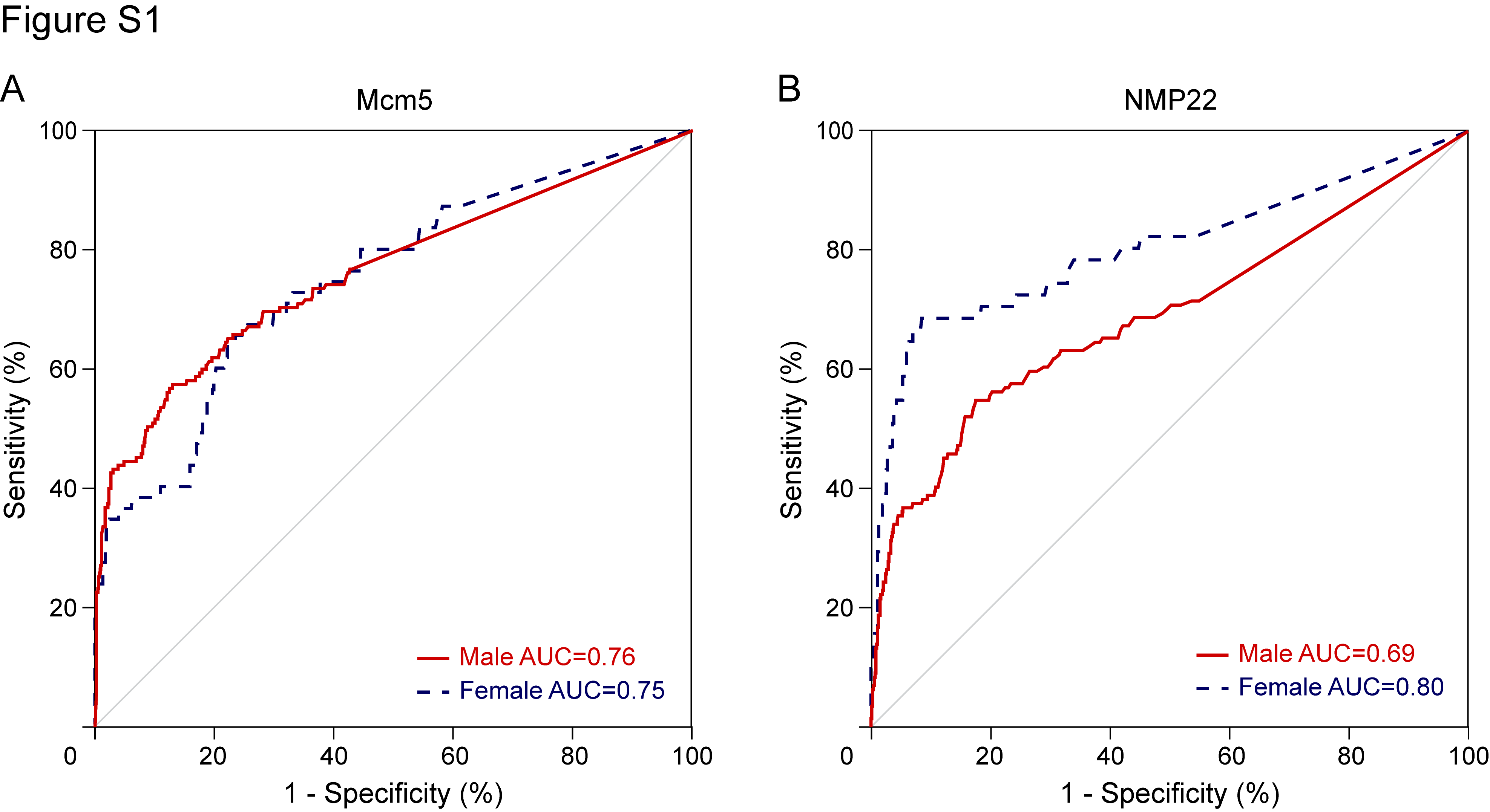

Supplement: Figure S1 — Receiver operating characteristics curves for the (A) Mcm5 and (B) NMP22 tests for detection of bladder cancer in male and female patients. (TIF) [file pone.0040305.s001.tif]
