# Supplementary material for: Bladder Cancer Diagnosis and Identification of Clinically Significant Disease by Combined Urinary Detection of Mcm5 and Nuclear Matrix Protein 22
Source: PLoS One. 2012 Jul 9;7(7):e40305. doi: 10.1371/journal.pone.0040305 (PMC3392249; doi:10.1371/journal.pone.0040305)
Supplement: Table S1 — Patient diagnoses. (PDF) [file pone.0040305.s002.pdf]

Table S1: Patient diagnoses

		n
Patients recruited		1677
Diagnosis	Malignancy of bladder/upper tract	222
	Clear normal	700
Other malignancies	Gynaecological	2
	Penile/testis	2
	Prostate cancer	35
	Renal cell carcinoma	17
Urolithiasis	Bladder calculus	15
	Renal calculus	99
	Vascular calcification in kidney	1
Other benign conditions	Benign prostatic hyperplasia	143
	Nephrological	45
	Prostatitis	14
	Urinary tract infection	273
Miscellaneous benign	Adult polycystic kidney disease	3
	Angiomyolipoma	5
	Atrophic/poorly functioning kidney	6
	Balanitis	3
	Bladder diverticulum	1
	Catheter related	3
	Chronic retention of urine	6
	Epididymo-orchitis	8
	Genitourinary tuberculosis	2
	Haemorrhagic cystitis	4
	Hydronephrosis	1
	Inverted papilloma	1
	Neurogenic/pathic bladder	3
	Oncocytoma	1
	Normal[a]	4
	Phimosis/meatal stenosis	3
	Pyelonephritis	8
	Pyelo-ureteric junction obstruction	3
	Pyogenic granuloma	1
	Radiation cystitis[b]	11
	Renal transplant	3
	Traumatic catheterisation	1
	Ureterocoele and retention of urine	1
	Urethral caruncle	2
	Urethral stricture	24
	Vascular malformation of kidney	1

[a] Patients had a history of bladder UCC (>5 years previously) or cyclophosphamide exposure
[b] Including one case with previous bladder UCC, four cases with previous prostate cancer
