# Supplementary material for: Bladder Cancer Diagnosis and Identification of Clinically Significant Disease by Combined Urinary Detection of Mcm5 and Nuclear Matrix Protein 22
Source: PLoS One. 2012 Jul 9;7(7):e40305. doi: 10.1371/journal.pone.0040305 (PMC3392249; doi:10.1371/journal.pone.0040305)
Supplement: Table S2 — True positive rate of Mcm5 and NMP22 tests and cytology, across stage, for bladder carcinoma detection. (PDF) [file pone.0040305.s003.pdf]

Table S2: True positive rate of Mcm5 and NMP22 tests and cytology, across stage, for bladder carcinoma detection

		Stage Ta/T1[a]		Stage T2/T3/T4[b]	
**Test**	**Cut-point**	**n**	**TPR, % (CI)**	**n**	**TPR, % (CI)**
Mcm5	1000-cell	163	77 (69-83)	38	92 (79-98)
	2150-cell	163	64 (57-72)	38	87 (72-96)
	8500-cell	163	34 (27-42)	38	74 (57-87)
NMP22	10 U/ml	154	47 (39-56)	34	79 (62-91)
Cytology		155	8 (5-14)	39	10 (3-24)

Abbreviations: CI, 95% confidence interval; TPR, true positive rate

[a] Non-muscle invasive

[b] Muscle invasive
