# Supplementary material for: Bladder Cancer Diagnosis and Identification of Clinically Significant Disease by Combined Urinary Detection of Mcm5 and Nuclear Matrix Protein 22
Source: PLoS One. 2012 Jul 9;7(7):e40305. doi: 10.1371/journal.pone.0040305 (PMC3392249; doi:10.1371/journal.pone.0040305)
Supplement: Table S3 — True positive rate of Mcm5 and NMP22 tests and cytology, across grade, for bladder carcinoma detection. (PDF) [file pone.0040305.s004.pdf]

Table S3: True positive rate of Mcm5 and NMP22 tests and cytology, across grade, for bladder carcinoma detection

		Grade 1		Grade 2		Grade 3/CIS	
**Test**	**Cut-point**	**n**	**TPR, % (CI)**	**n**	**TPR, % (CI)**	**n**	**TPR, % (CI)**
Mcm5	1000-cell	23	61 (39-80)	123	77 (69-84)	63	92 (82-97)
	2150-cell	23	48 (27-69)	123	63 (54-72)	63	89 (78-95)
	8500-cell	23	13 (3-34)	123	33 (25-42)	63	71 (59-82)
NMP22	10 U/ml	25	20 (7-41)	112	51 (41-60)	58	72 (59-83)
Cytology		23	4 (1-22)	117	7 (3-13)	62	15 (7-26)

Abbreviations: CI, 95% confidence interval; CIS, carcinoma in situ; TPR, true positive rate
