# Supplementary material for: Bladder Cancer Diagnosis and Identification of Clinically Significant Disease by Combined Urinary Detection of Mcm5 and Nuclear Matrix Protein 22
Source: PLoS One. 2012 Jul 9;7(7):e40305. doi: 10.1371/journal.pone.0040305 (PMC3392249; doi:10.1371/journal.pone.0040305)
Supplement: Table S4 — Comparison of Mcm5 and NMP22 test performance in male and female patients. (PDF) [file pone.0040305.s005.pdf]

Table S4: Comparison of Mcm5 and NMP22 test performance in male and female patients

	Male		Female		
**Test**	**n**	**AUC (CI)**	**n**	**AUC (CI)**	**P**[a]
Mcm5[b]	983	0.76 (0.72-0.81)	581	0.75 (0.68-0.82)	0.76
NMP22[c]	874	0.69 (0.64-0.75)	522	0.80 (0.72-0.88)	0.025

Abbreviations: AUC, area under the curve; CI, 95% confidence interval

[a] Chi-squared test comparing AUC values for males vs females

[b] Based on 155 and 828 male patients and 55 and 526 female patients respectively with and without UCC

[c] Based on 144 and 730 male patients and 51 and 471 female patients respectively with and without UCC
