# Supplementary material for: Bladder Cancer Diagnosis and Identification of Clinically Significant Disease by Combined Urinary Detection of Mcm5 and Nuclear Matrix Protein 22
Source: PLoS One. 2012 Jul 9;7(7):e40305. doi: 10.1371/journal.pone.0040305 (PMC3392249; doi:10.1371/journal.pone.0040305)
Supplement: Table S5 — True and false negative rates of the Mcm5 and NMP22 tests, by tumour grade and stage. (PDF) [file pone.0040305.s006.pdf]

Table S5: True and false negative rates of the Mcm5 and NMP22 tests, by tumour grade and stage

	TNR, % (CI)		FNR, % (CI)											
**Cut-point**	**n**	**Normal**	**n**	**Grade 1**	**n**	**Grade 2**	**n**	**Grade 3**	**n**	**Stage pTa**	**n**	**Stage pT1**	**n**	**Stage ≥pT2**
Mcm5														
1000-cell	1354	50 (47-53)	23	39 (20-61)	123	23 (16-31)	55	7 (2-18)	115	31 (23-41)	48	4 (1-14)	38	8 (2-21)
2150-cell	1354	69 (66-71)	23	52 (31-73)	123	37 (28-46)	55	11 (4-22)	115	46 (38-56)	48	10 (3-23)	38	13 (4-28)
8500-cell	1354	95 (94-96)	23	87 (66-97)	123	67 (58-75)	55	27 (16-41)	115	81 (72-88)	48	29 (17-44)	38	26 (13-43)
NMP22														
10 U/ml	1201	84 (82-86)	25	80 (59-93)	112	49 (40-59)	51	25 (14-40)	109	62 (53-71)	45	29 (16-44)	34	21 (9-38)

Abbreviations: CI, 95% confidence interval; FNR, false negative rate; TNR, true negative rate (specificity)
